# Molecular Characterization of Lumpy Skin Disease Virus in Bulgaria: Phylogenetic Insights Based on the RPO30 Gene

**DOI:** 10.3390/vetsci13101086

**Published:** 2026-10-14

**Authors:** Gabriela Goujgoulova, Koycho Koev, Georgi Stoimenov

**Affiliations:** 1 Risk Assessment Center on Food Chain, 1000 Sofia, Bulgaria; gvgoujgoulova@abv.bg; 2 Department of Veterinary Microbiology, Infectious and Parasitic Diseases, Faculty of Veterinary Medicine, Trakia University, 6000 Stara Zagora, Bulgaria; 3 Department of Infectious Pathology and Food Hygiene, Faculty of Veterinary Medicine, University of Forestry, 1796 Sofia, Bulgaria; georgi.stoimenov.vm@gmail.com

**Keywords:** capripoxvirus, RPO30 gene, Bulgaria

## Abstract

Capripoxviruses are important viral pathogens of domestic ruminants and include lumpy skin disease virus (LSDV), sheeppox virus (SPPV), and goatpox virus (GTPV). In recent years, the spread of LSDV into Southeastern Europe has renewed interest in its molecular epidemiology, particularly in Balkan countries. This study analyzed partial RPO30 gene sequences obtained from clinical samples collected during the 2016 LSDV outbreak in Bulgaria. Viral DNA was detected by real-time PCR, and RPO30 amplicons obtained by conventional PCR were subjected to Sanger sequencing. The obtained sequences were compared with representative LSDV reference sequences retrieved from GenBank, and phylogenetic relationships were reconstructed using the Maximum Likelihood method. The five Bulgarian sequences included in the final analysis were positioned near Balkan and Neethling-type LSDV reference sequences and showed high sequence similarity within the analyzed partial RPO30 region. Single nucleotide polymorphisms enabled differentiation among the analyzed sequences and revealed limited variation within the Bulgarian sequence set. Haplotype analysis identified 10 haplotypes across the final 30-sequence dataset; however, all haplotype, SNP, distance-based, and phylogenetic results derive from the same partial RPO30 marker and were interpreted accordingly. The data provide additional information on the molecular characteristics of the analyzed Bulgarian LSDV sequences from the 2016 outbreak, while emphasizing the need for higher-resolution genomic approaches for recombinant or vaccine-like strain characterization and fine-scale epidemiological inference.

## Simple Summary

Capripoxviruses cause serious diseases in cattle, sheep and goats, and lumpy skin disease remains a concern for Southeastern Europe. This short communication examines Bulgarian LSDV sequences obtained from clinical samples collected during the 2016 outbreaks using partial RPO30 gene sequencing. The five Bulgarian sequences retained for the final analysis showed high sequence similarity to Balkan and Neethling-type LSDV reference sequences, with limited RPO30 variation within the analyzed sequence set. These findings support the usefulness of RPO30-based comparative molecular characterization and regional sequence comparison, while also indicating that whole-genome sequencing would be required for higher-resolution outbreak tracing.

## 1. Introduction

Viruses of the genus Capripoxvirus (family Poxviridae) are responsible for economically significant diseases in domestic ruminants worldwide. This genus includes lumpy skin disease virus (LSDV), sheeppox virus (SPPV), and goatpox virus (GTPV), which are closely related at the genomic level but differ in host specificity and epidemiological distribution [1,2]. Infected animals typically develop fever, generalized skin nodules, lymphadenopathy, and, in severe cases, significant production losses.

Lumpy skin disease has gained increasing global attention over the past decade due to its rapid geographic expansion beyond its traditional endemic regions in Africa. Since 2012, LSDV has spread into the Middle East, Southeastern Europe, and parts of Asia, affecting previously disease-free regions [3,4]. The Balkan Peninsula, including Bulgaria, was affected during outbreaks reported in 2015–2016. More recently, the emergence of recombinant and vaccine-like LSDV strains in several regions has further complicated epidemiological investigations, as these variants may exhibit genetic profiles that are not readily distinguishable without detailed molecular analysis.

Sheeppox and goatpox remain important endemic diseases in many regions and continue to cause substantial economic losses in small ruminant populations. Due to the high genetic similarity among capripoxviruses, reliable differentiation between LSDV, SPPV, and GTPV cannot be achieved using clinical or conventional diagnostic approaches alone and requires molecular methods [1].

Molecular characterization of capripoxviruses has become a key tool in understanding their genetic diversity and epidemiological relationships. Several genomic regions have been used for this purpose, including the RPO30 gene, which encodes a subunit of the viral RNA polymerase. This gene is generally conserved but contains sufficient variation to allow discrimination between species and, to some extent, between strains [5]. Although several molecular markers have been proposed for capripoxvirus characterization, the RPO30 gene remains one of the most widely used targets due to its balance between conservation and discriminatory capacity, particularly in studies where whole-genome sequencing is not available. Previous studies have demonstrated its applicability in phylogenetic analyses and in the identification of specific nucleotide polymorphisms associated with different capripoxvirus lineages [3]. Recent full-length RPO30 sequence analysis across capripoxviruses further demonstrated the high conservation of this marker while identifying species-specific nucleotide signatures that support its continued use for comparative molecular characterization [6].

Despite the epidemiological importance of lumpy skin disease in Bulgaria, molecular data on circulating strains remain limited, particularly in the context of comparative phylogenetic analyses with regional and global isolates.

The aim of the present study was to characterize partial RPO30 gene sequences generated from clinical samples collected during the 2016 LSDV outbreaks in Bulgaria and to assess their phylogenetic relationships with representative LSDV sequences available in public databases.

## 2. Materials and Methods

### 2.1. Sample Collection

Eight clinical samples were collected from cattle exhibiting lesions consistent with lumpy skin disease during outbreaks in Bulgaria in April–May 2016. Sampling sites included the Plovdiv, Haskovo, Stara Zagora, Kyustendil, and Blagoevgrad regions. Specimens comprised blood, skin nodules, and nasal swabs collected from naturally infected animals. Seven samples yielded RPO30 sequence data, and five Bulgarian field sequences were retained for the final phylogenetic analysis. Clinical sample metadata and sequencing outcomes are summarized in Supplementary Table S1. The five Bulgarian field sequences retained for the final phylogenetic analysis were generated from four blood samples (102, 115, 116, and 119) and one nasal-discharge sample (74). All samples were transported under refrigerated conditions and stored at −20 °C until processing. The five samples represented four geographical regions: two originated from the Plovdiv region (102 and 116), while one sample each originated from the Haskovo (115), Kyustendil (74), and Blagoevgrad (119) regions. Detailed herd-level epidemiological information was not available to establish epidemiological links among the sampled animals or outbreaks. At the time of sampling (April–May 2016), LSDV vaccination had not yet been introduced in Bulgaria; therefore, the sampled cattle had not been vaccinated against LSDV. Characteristic skin nodules were documented among the clinical findings where this information was available. Complete information on individual animal age, sex, and standardized clinical severity was not available for all sampled animals; consequently, these variables were not included in quantitative epidemiological interpretation. No formal a priori sample-size calculation could be documented for the available archived sample set; the study therefore represents molecular characterization of the available diagnostic specimens rather than a statistically designed prevalence or population-genetic survey.

### 2.2. DNA Extraction and Molecular Detection

Viral DNA was extracted from clinical specimens using the DNeasy Blood & Tissue Kit (Qiagen, Hilden, Germany; Cat. No. 69504), according to the manufacturer’s instructions. Capripoxvirus DNA was detected using a TaqMan real-time PCR assay targeting ORF074, encoding the P32 protein, with primers CaPV-074F1 (5′-AAAACGGTATATGGAATAGAGTTGGAA-3′) and CaPV-074R1 (5′-AAATGAAACCAATGGATGGGATA-3′) and the FAM-labelled minor-groove-binder probe CaPV-074P1 (5′-FAM-TGGCTCATAGATTTCCT-MGBNFQ-3′), as described by Bowden et al. [7]. Reactions were performed using the QuantiFast Probe PCR +ROX Vial Kit (Qiagen, Hilden, Germany; Cat. No. 204354) on an Mx3005P Multiplex Quantitative PCR System (Stratagene, La Jolla, CA, USA). Amplification consisted of an initial activation step at 95 °C for 10 min, followed by 45 cycles of denaturation at 95 °C for 15 s and annealing/extension at 60 °C for 60 s. Samples exhibiting characteristic amplification curves with cycle threshold (Ct) values < 39 were considered positive.

Samples yielding positive real-time PCR results were subsequently subjected to conventional RPO30 PCR and bidirectional Sanger sequencing for molecular characterization. No additional GPCR- or ORF103-targeting assays were used for confirmatory testing.

### 2.3. PCR Amplification and Sequencing of RPO30 Gene

The RPO30 coding region was amplified as two overlapping conventional PCR fragments using the primer pairs described by Gelaye et al. [8]. The first fragment, with an expected amplicon size of 554 bp, was amplified using CpRPO30-OL1F (5′-CAGCTGTTTGTTTACATTTGATTTTT-3′) and CpRPO30-OL1R (5′-TCGTATAGAAACAAGCCTTTAATAGA-3′). The second fragment, with an expected amplicon size of 520 bp, was amplified using CpRPO30-OL2F (5′-TTTGAACACATTTTATTCCAAAAAG-3′) and CpRPO30-OL2R (5′-AACCTACATGCATAAACAGAAGC-3′). The stated fragment sizes refer to the expected PCR amplicons generated by the individual primer pairs and should not be interpreted as the final length of the assembled sequence or as the retained sequence coverage represented in the final alignment.

Each PCR was performed in a final reaction volume of 25 µL containing 1× PCR buffer, 0.2 mM of each dNTP, 500 nM of each primer, 2.5 U of Taq DNA polymerase (Qiagen, Hilden, Germany), and 5 µL of extracted DNA. Amplification comprised an initial denaturation at 95 °C for 4 min, followed by 40 cycles of denaturation at 95 °C for 30 s, annealing at 55 °C for 30 s, and extension at 72 °C for 45 s, with a final extension at 72 °C for 7 min. PCR products were examined by electrophoresis on a 1.5% agarose gel, and amplicons of the expected sizes (554 bp and 520 bp) were purified prior to sequencing, following the established overlapping-RPO30 workflow [9].

Bidirectional Sanger sequencing was performed by LGC Genomics (Berlin, Germany). Each overlapping RPO30 fragment was sequenced from both the forward and reverse directions using the corresponding amplification primers, thereby providing complementary sequence coverage across both amplicons and their overlapping region [9].

### 2.4. Sequence Processing and Dataset Compilation

Raw Sanger chromatograms were edited and assembled using the Staden software package version 2.0.0b8 (Pregap4/Gap4) [9]. Forward and reverse chromatograms were visually inspected for peak quality, background noise, and read concordance. No fixed numerical Phred quality-score threshold was applied; low-quality terminal bases and positions with unresolved or discordant nucleotide calls were excluded during sequence editing rather than assigned retrospectively. Primer-derived terminal sequences were removed during sequence editing before construction of the final consensus sequences. For each amplicon, the quality-filtered complementary forward and reverse reads were assembled into a consensus sequence, and ambiguous nucleotide calls were accepted only when supported by interpretable complementary trace data. The consensus sequences generated from the two overlapping RPO30 amplicons were subsequently joined through their shared region to generate an RPO30 consensus sequence for each clinical sample. The assembled sequences were re-inspected, and terminal low-quality sequence was removed before alignment. Because retained terminal sequence coverage differed among the Bulgarian sequences, positions without retained sequence data were represented as missing/gap states in the final alignment rather than being treated as nucleotide differences. Only quality-controlled nucleotide calls within the homologous RPO30 alignment were used for SNP identification, genetic-distance calculations, haplotype and sequence-based cluster analyses, and phylogenetic reconstruction. Sequence differences located exclusively outside the region shared by all five Bulgarian sequences were not used as sole evidence for distinguishing sequence profiles. Of the seven successfully sequenced clinical samples, five Bulgarian sequences were retained in the final phylogenetic dataset. In the archived final aligned FASTA dataset, these five retained sequences contain the following numbers of non-gap nucleotide characters: sample 74 (1127), sample 102 (832), sample 115 (1127; including one ambiguous N), sample 116 (1129), and sample 119 (1000). These values describe retained alignment coverage in the archived analysis file and are reported separately from the expected PCR product sizes.

For comparative analysis, 25 reference RPO30 sequences of lumpy skin disease virus (LSDV) were retrieved from the NCBI GenBank database and analyzed together with the five Bulgarian sequences retained in the final dataset. Reference sequences were selected to provide regional and broader temporal context for the Bulgarian 2016 outbreak sequences, with emphasis on strains from the Balkan Peninsula and neighboring regions affected during the 2015–2017 epidemic period, together with selected geographically and temporally more distant LSDV sequences for broader comparative context. The final comparative dataset therefore comprised 30 RPO30 sequences: five Bulgarian sequences generated in the present study and 25 GenBank reference sequences. Accession number, strain/isolate designation, virus species, host, country of origin, year of collection, and source-supported strain status are provided in Supplementary Table S2. Where field, vaccine-related, or recombinant status could not be established from the corresponding GenBank record or associated publication, the status was recorded as not reported (NR). Duplicate entries were removed, and all sequences were standardized to the common homologous RPO30 region prior to alignment.

### 2.5. Sequence Alignment and Phylogenetic Analysis

Multiple sequence alignment was performed using the MUSCLE algorithm implemented in MEGA version 11. Alignments were manually inspected and adjusted where necessary. After standardization to the common homologous RPO30 region, the final alignment comprised 1131 nucleotide positions and 30 sequences: five Bulgarian field sequences generated in this study and 25 reference sequences retrieved from GenBank. The final multiple-sequence alignment used for the comparative, phylogenetic, genetic-distance, haplotype, and SNP analyses is provided as Supplementary File S1 in aligned FASTA format. Because the validated SNP positions are reported as alignment-column coordinates and the archived dataset does not provide an unambiguous codon/reference-coordinate mapping for every retained sequence, synonymous versus nonsynonymous consequences and overlap with RPO30 primer-binding sites were not inferred from these alignment-relative positions.

Phylogenetic relationships were inferred using the Maximum Likelihood method implemented in MEGA version 11 [10]. Model selection was performed according to the Akaike Information Criterion (AIC), and the Tamura–Nei (TN93) nucleotide substitution model was selected as the best-fitting model for the analyzed alignment. No partitioning scheme was applied because the analysis was based on a single partial RPO30 locus. Statistical support for tree topology was evaluated using 1000 nonparametric bootstrap replicates [11]. Bootstrap support >70% was regarded as well supported, whereas support <50% was considered weak and was not used to define robust phylogenetic relationships. No additional branch-support statistics, including SH-aLRT or aBayes, were calculated. The resulting Maximum Likelihood tree was visualized and annotated using FigTree version 1.4.4 [12]. For the integrated analysis, a complementary neighbor-joining tree was reconstructed from pairwise nucleotide p-distances calculated with pairwise deletion of gap or ambiguous states. Haplotype frequencies, sequence membership, and sequence-based cluster designations are reported in Supplementary Table S3. Given the short shared RPO30 region and the limited number of sequences, no LOESS or other smoothing-based SNP-density analysis was retained; nucleotide variation was summarized directly at individual polymorphic sites. The neighbor-joining analysis was used only as a complementary distance-based visualization of the same partial RPO30 dataset and was not interpreted as an independent reconstruction of evolutionary history.

### 2.6. Haplotype and Sequence-Based Cluster Analysis

Haplotype analysis was performed in R version 4.4.3 using the ape package version 5.8-1. Variable nucleotide positions in the final aligned RPO30 dataset were identified using seg.sites(). For each sequence, nucleotide states at the variable positions were concatenated to generate a sequence-specific SNP profile, and sequences with identical profiles across the analyzed alignment were assigned to the same haplotype. Ten unique haplotypes were identified in the final 30-sequence dataset and were numbered sequentially H1–H10 according to the order in which unique SNP profiles first occurred. Haplotype frequencies and the sequence identifiers assigned to each haplotype are provided in Supplementary Table S3. Genetic distances were additionally calculated using dist.dna(), followed by average-linkage hierarchical clustering with hclust(method = “average”); the dendrogram was partitioned into three sequence-based groups using cutree(k = 3), designated C1–C3. These groups were used as descriptive sequence-based clusters and were not treated as independently supported phylogenetic clades. A separate haplotype-network analysis was not performed; relationships among sequence variants were summarized by haplotype membership, frequency, sequence-based clustering, and the phylogenetic analyses described above. Because the phylogenetic, haplotype, SNP, and distance-based analyses were all derived from the same partial RPO30 alignment, concordance among these analyses was interpreted as internal consistency within this marker rather than as independent evidence of distinct evolutionary lineages.

## 3. Results

**Figure 1:**
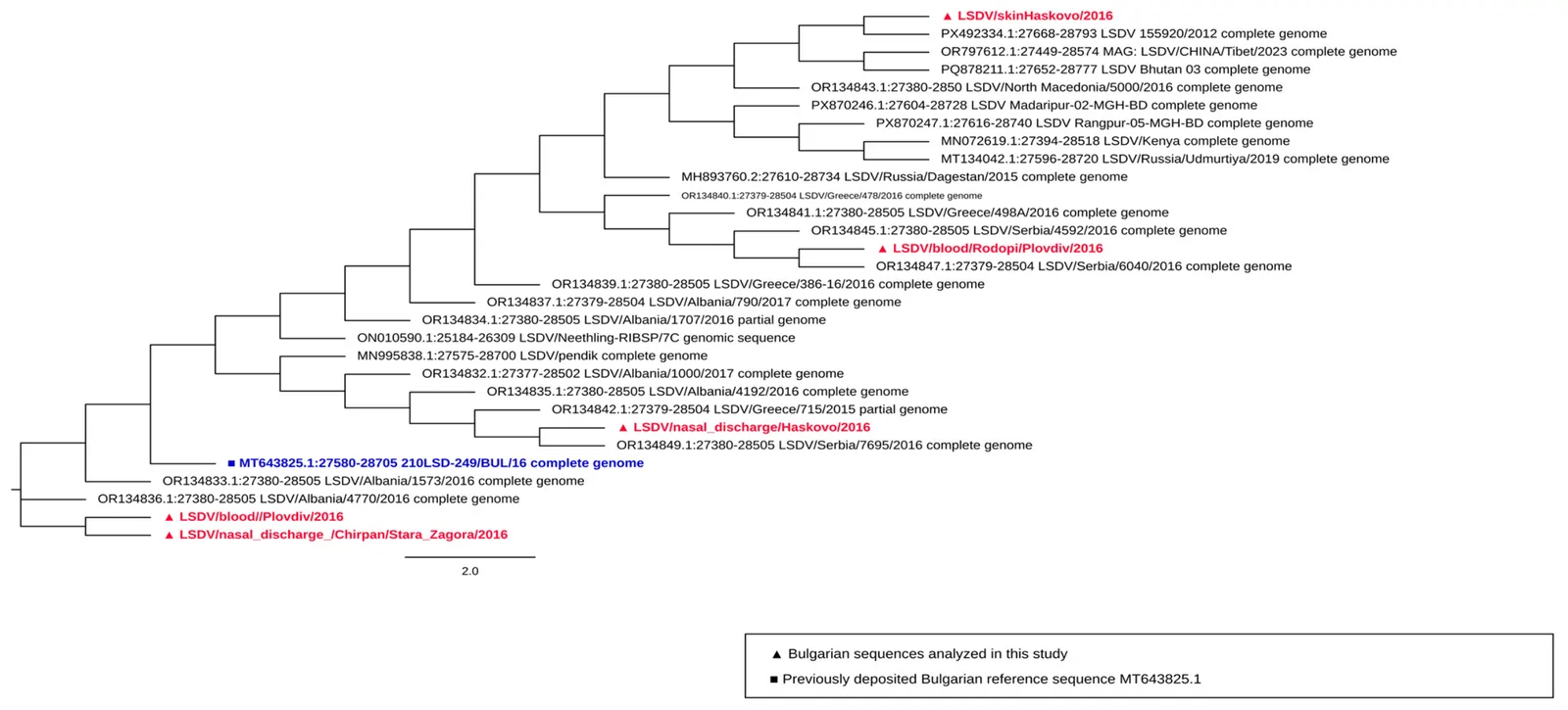
Phylogenetic analysis of LSDV RPO30 gene sequences.

The final RPO30 alignment comprised 30 sequences and 1131 nucleotide positions, including five Bulgarian field sequences generated in this study and 25 reference LSDV sequences retrieved from GenBank (Supplementary Table S2). Maximum Likelihood analysis was performed using the Tamura–Nei (TN93) nucleotide substitution model with 1000 bootstrap replicates. Only well-supported internal relationships were used for phylogenetic interpretation, whereas weakly supported relationships were treated descriptively (Figure 1).

Maximum Likelihood tree based on partial RPO30 gene sequences using the Tamura–Nei (TN93) nucleotide substitution model. Bootstrap support was assessed using 1000 replicates; values > 70% were regarded as well supported, whereas values < 50% indicated weak support. The five newly generated Bulgarian RPO30 sequences correspond to samples 74, 102, 115, 116, and 119 and are indicated by filled triangles (▲). MT643825.1 (LSDV_BUL2016) is a previously deposited Bulgarian LSDV reference sequence retrieved from GenBank and is indicated by a filled square (■) for comparative purposes. The shape markers provide an additional distinction independent of color for grayscale reproduction.

In the Maximum Likelihood tree, the five Bulgarian sequences were positioned in proximity to several Balkan and Neethling-type LSDV reference sequences. Quantitative comparison with the Neethling-RIBSP/7C reference sequence (ON010590.1), using pairwise deletion of gap or ambiguous states, showed nucleotide identities of 99.29% for sample 74, 99.04% for sample 102, 99.38% for sample 115, 99.47% for sample 116, and 98.10% for sample 119, corresponding to pairwise p-distances of 0.0071, 0.0096, 0.0062, 0.0053, and 0.0190, respectively. Among the five Bulgarian sequences, pairwise nucleotide identity ranged from 97.70% to 99.56%, corresponding to p-distances of 0.0044–0.0230.

Across the shared quality-controlled RPO30 region of the five Bulgarian sequences, 16 validated polymorphic nucleotide positions were identified. The exact alignment positions and nucleotide states are provided in Supplementary Table S4. These positions were 77, 81, 82, 84, 85, 86, 105, 160, 273, 372, 452, 561, 667, 798, 801, and 803 in the multiple-sequence alignment.

**Figure 2:**
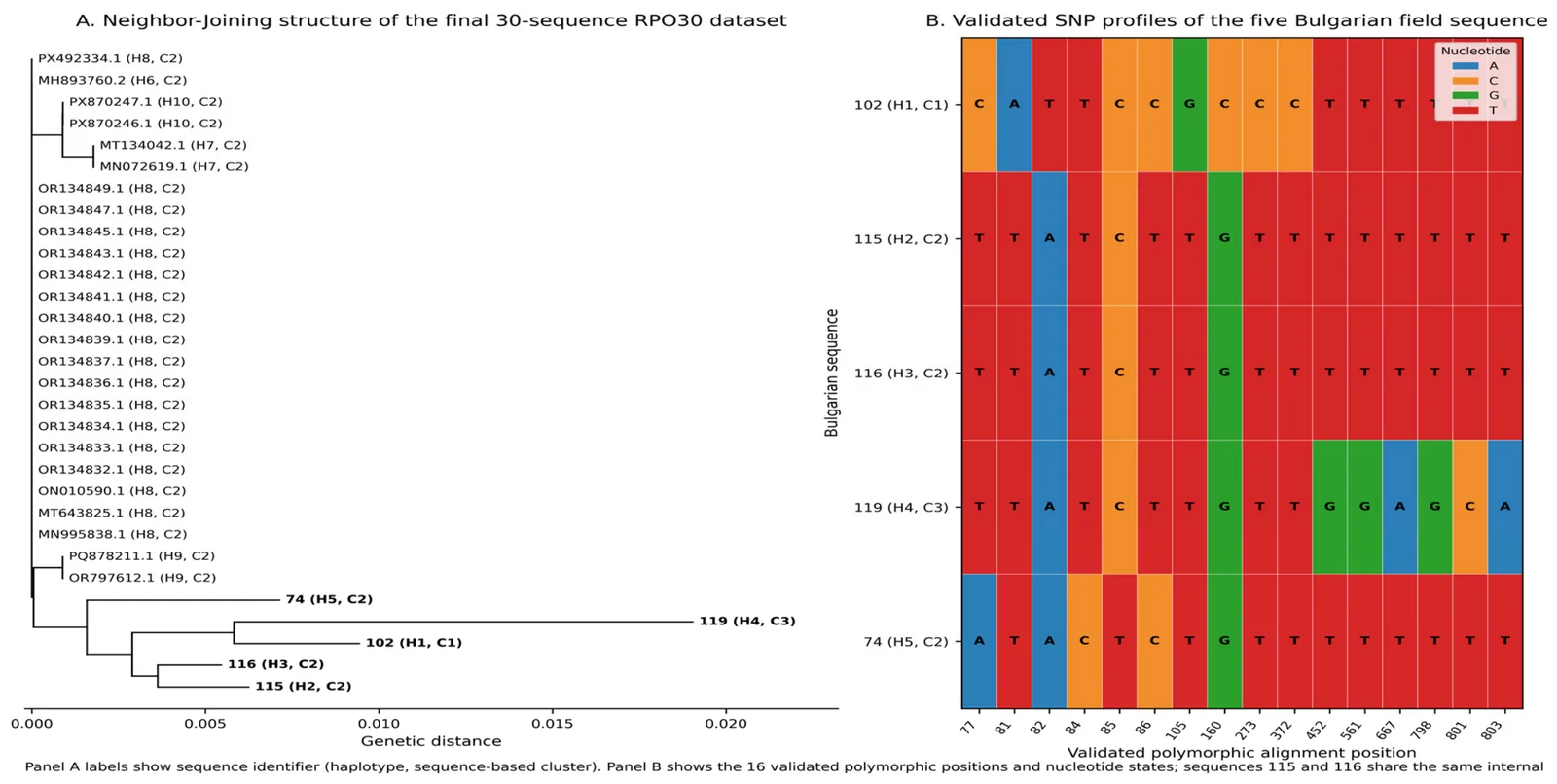
Distance-based sequence structure, validated SNP variation, and haplotype assignment across the analyzed RPO30 dataset. (**A**) Complementary neighbor-joining tree of the final 30-sequence RPO30 dataset reconstructed from pairwise nucleotide p-distances. Each terminal label reports the sequence identifier together with its haplotype designation and sequence-based cluster (C1–C3), allowing the sequence identifiers and haplotype assignments to be read directly. (**B**) SNP matrix of the five Bulgarian field sequences (74, 102, 115, 116, and 119) at the 16 validated polymorphic positions within their shared quality-aligned RPO30 region. Position numbers refer to columns in the final 1131-position multiple-sequence alignment, with position 1 corresponding to the first alignment column; they are not coordinates of a single reference sequence. Only nucleotide substitutions (A, C, G, and T) are displayed; insertion/deletion sites are not represented in the matrix. Haplotype and cluster designations are shown with the isolate labels.

SNP variation was evaluated together with the complementary neighbor-joining structure, haplotype assignments, and sequence-based clusters. Figure 2 displays all 30 sequences in the final comparative dataset with their haplotype and cluster designations and shows the nucleotide states of the five Bulgarian field sequences at the validated polymorphic positions (Supplementary Tables S3 and S4).

Samples 74, 102, and 119 displayed distinct internal SNP profiles, whereas sequences 115 and 116 had identical nucleotide states at all 16 validated internal polymorphic positions within the shared quality-aligned region (Supplementary Table S4).

Sequences 115 and 116 showed identical nucleotide states at all 16 validated internal polymorphic positions despite their distinct full-alignment haplotype assignments. Complete haplotype and cluster assignments and the validated nucleotide states are provided in Supplementary Tables S3 and S4.

**Figure 3:**
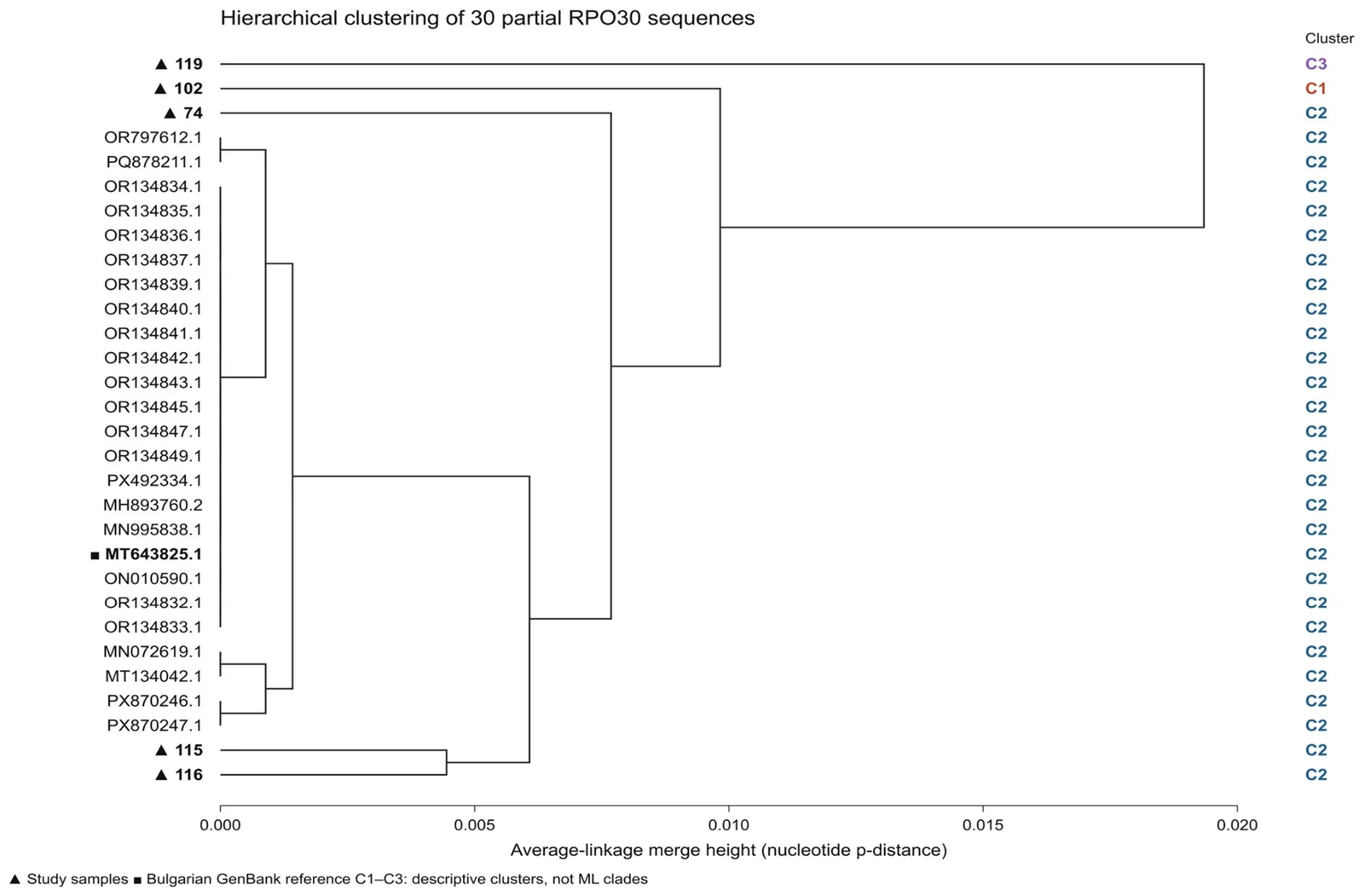
Average-linkage hierarchical clustering of the final 30-sequence partial RPO30 alignment. Pairwise nucleotide p-distances were calculated using pairwise deletion of gaps and ambiguous nucleotide states. Merge heights represent average distances between clusters. The dendrogram was partitioned into three descriptive clusters (C1–C3): C1 comprises sample 102; C3 comprises sample 119; C2 comprises the remaining 28 sequences. Triangles identify the five study samples; the square identifies the previously deposited Bulgarian reference MT643825.1. Sequence identifiers correspond to Supplementary Tables S1–S3. These clusters are not Maximum Likelihood clades.

Figure 3 summarizes a separate descriptive analysis based on average-linkage hierarchical clustering of pairwise nucleotide p-distances rather than a second phylogenetic reconstruction. Accordingly, differences in branching order between Figure 1 and Figure 3 reflect the different analytical procedures and are not interpreted as conflicting phylogenetic results.

Cluster C1 contained only Bulgarian sample 102, whereas C3 contained only Bulgarian sample 119. Cluster C2 contained Bulgarian samples 74, 115, and 116 together with all 25 GenBank reference sequences, including the previously deposited Bulgarian sequence MT643825.1 and the Neethling-RIBSP/7C reference ON010590.1 (Supplementary Table S3). Because C1–C3 were derived from hierarchical clustering rather than from branches of the Maximum Likelihood tree, bootstrap support values are not applicable to these cluster assignments; bootstrap support is reported separately for the Maximum Likelihood topology in Figure 1.

Haplotype analysis of the final 30-sequence RPO30 alignment identified 10 haplotypes (H1–H10). H1–H6 each contained one sequence, H7 contained two sequences, H8 contained 18 sequences, H9 contained two sequences, and H10 contained two sequences. The five Bulgarian sequences were assigned as follows: sample 102 to H1, sample 115 to H2, sample 116 to H3, sample 119 to H4, and sample 74 to H5; complete haplotype frequencies, sequence membership, and corresponding sequence-based clusters are provided in Supplementary Table S3.

Validation restricted to the shared high-quality internal region resolved four distinguishable SNP profiles among the five Bulgarian sequences: samples 74, 102, and 119 retained distinct profiles, whereas sequences 115 and 116 were identical across all 16 validated internal polymorphic positions (Supplementary Table S4).

MT643825.1 was included as a previously deposited Bulgarian LSDV reference sequence for comparison with the five newly generated Bulgarian RPO30 sequences corresponding to samples 74, 102, 115, 116, and 119. MT643825.1 is therefore distinct from the five sequences generated in the present study. Reference-sequence accession numbers and associated metadata are listed in Supplementary Table S2.

## 4. Discussion

Molecular characterization of capripoxviruses remains important for investigating their epidemiology, particularly in regions affected by outbreaks such as Southeastern Europe. In the present study, analysis of the RPO30 gene confirmed a high level of sequence conservation among the examined sequences, consistent with previous reports describing the conserved nature of this marker [2,5].

Despite this overall conservation, single nucleotide polymorphisms (SNPs) enabled differentiation among the analyzed LSDV sequences and detection of minor variation among closely related sequences. Previous studies have demonstrated that limited sequence variability within the RPO30 gene can support phylogenetic discrimination among capripoxvirus species [4,5]. The distinct SNP patterns observed in the present dataset further support the utility of this gene as a marker for comparative analyses.

Phylogenetic reconstruction positioned the five Bulgarian sequences near Balkan and Neethling-type reference sequences, consistent with high sequence similarity within the partial RPO30 region rather than with evidence for a distinct sublineage. Because the final comparative dataset contained only LSDV sequences, the present phylogenetic analysis was not designed to evaluate interspecies discrimination among LSDV, SPPV, and GTPV. The concordance between sequence-based grouping, haplotype assignment, and SNP patterns indicates structured sequence variation within the analyzed dataset, but these descriptive groups should not be equated with independently supported evolutionary lineages.

The limited RPO30 variability observed among the five Bulgarian sequences indicates a relatively homogeneous sequence profile within the analyzed dataset. Their proximity to Balkan and Neethling-type references is consistent with previously reported regional sequence relationships during the 2015–2017 outbreak period. Nevertheless, given the small sample size, variable bootstrap support, and limited epidemiological metadata, these findings should not be extrapolated to the overall genetic diversity of LSDV circulating in Bulgaria during the 2016 outbreak. The available sequence and epidemiological data are insufficient to infer a common source, the number of introduction events, herd-to-herd transmission, or specific transmission chains. Accordingly, the observed similarity among the five Bulgarian sequences should be interpreted only as sequence similarity within the partial RPO30 region and not as evidence of a shared epidemiological source or direct local transmission. After exclusion of terminal-only differences from interpretation, four distinguishable internal SNP profiles were observed among the five Bulgarian sequences, with sequences 115 and 116 remaining identical across the shared aligned region.

However, the use of a single genetic marker such as the RPO30 gene has inherent limitations. While suitable for species differentiation and basic phylogenetic analysis, it lacks the resolution required for detailed genotyping or for the detection of recombinant strains. This limitation is particularly relevant given the recent emergence of recombinant and vaccine-like LSDV strains in Eurasia, which often exhibit complex genomic profiles that cannot be reliably identified using single-gene approaches [13,14]. Recent whole-genome sequencing of outbreak-associated LSDV has revealed extensive genome-wide SNP and indel variation and genetically distinct circulating groups, illustrating the additional resolution available beyond a single partial marker [15]. These genome-level observations are cited here only to illustrate the limitations of the present partial-marker approach and should not be interpreted as findings derived from the Bulgarian RPO30 dataset.

In this context, the absence of whole-genome sequence data represents a limitation of the present study. Accordingly, the present study does not permit conclusions regarding genome-wide variation, recombinant or vaccine-like genomic profiles, or high-resolution genomic epidemiology. In addition, the limited availability of publicly accessible sequence data from more recent outbreaks in Bulgaria restricts detailed temporal comparisons. Recent phylogenomic and phylogeographic analyses have likewise identified multiple genetically structured LSDV clusters and haplotypes across geographic regions, reinforcing the value of genome-scale surveillance for interpreting contemporary viral diversity [16]. Future studies should therefore focus on expanded molecular surveillance incorporating whole-genome sequencing or multilocus approaches, which would enable more comprehensive characterization of viral diversity, improved detection of recombinant strains, and more accurate reconstruction of transmission pathways.

## 5. Conclusions

The present study confirms that the five Bulgarian RPO30 sequences included in the final analysis are closely related to previously reported Balkan and Neethling-type LSDV reference sequences. After exclusion of terminal-only sequence differences, four distinguishable internal SNP profiles were identified within the shared aligned region, with sequences 115 and 116 showing an identical internal profile. These findings support the use of partial RPO30 sequencing for comparative molecular characterization of LSDV sequences, while also emphasizing its limited resolution for fine-scale epidemiological inference and its inability to support identification of recombinant or vaccine-like genomic profiles. Because of the small sample size and limited epidemiological metadata, these findings characterize the analyzed sequences and should not be interpreted as representative of the overall genetic diversity or transmission dynamics of the 2016 Bulgarian LSD outbreak.

## Data Availability

The original contributions presented in this study are included in the article/Supplementary Materials. Further inquiries can be directed to the corresponding author. Clinical sample metadata and sequencing outcomes are provided in Supplementary Table S1. Accession numbers and related metadata of the reference sequences used for the comparative and phylogenetic analyses are provided in Supplementary Table S2. Haplotype and sequence-based cluster assignments are provided in Supplementary Table S3. The exact polymorphic alignment positions and nucleotide states used to validate the Bulgarian SNP profiles are provided in Supplementary Table S4. The final 30-sequence RPO30 multiple-sequence alignment is provided as Supplementary File S1 in aligned FASTA format.

## Supplementary Materials

The following supporting information can be downloaded at: https://www.mdpi.com/article/10.3390/vetsci13101086/s1, Table S1: Clinical samples collected during the 2016 LSD outbreak in Bulgaria and their sequencing outcome; Table S2: Annotated dataset of LSDV RPO30 reference sequences used in the comparative analysis; Table S3: Haplotype and sequence-based cluster assignment of LSDV RPO30 sequences included in this study; Table S4: Polymorphic nucleotide positions within the shared aligned RPO30 region of the five Bulgarian LSDV sequences; Supplementary File S1: Final aligned FASTA file containing the 30 RPO30 sequences used for the comparative, phylogenetic, genetic-distance, haplotype, and SNP analyses.
