# Supplementary material for: Molecular Characterization of Lumpy Skin Disease Virus in Bulgaria: Phylogenetic Insights Based on the RPO30 Gene
Source: Vet Sci. 2026 Oct 14;13(10):1086. doi: 10.3390/vetsci13101086 (PMC13709801; doi:10.3390/vetsci13101086)
Supplement: Supplementary file 1 [file vetsci-13-01086-s001.zip › Supplementary_Tables_S1-S4_Final.pdf]

Table S1. Clinical samples collected during the 2016 LSD outbreak in Bulgaria and their sequencing outcome.

**Sample ID**	**Date**	**Location**	**Sample type**	**Clinical signs**	Host	**Sequenced**	Non-gap nucleotide characters in final aligned FASTA
no0	12 Apr 2016	Voden, Haskovo	Skin nodule	skin nodules	Cattle	Yes	Not retained
no1	12 Apr 2016	Chirpan, Stara Zagora	Nasal swab	skin nodules	Cattle	Yes	Not retained
74	29 Apr 2016	Buranovo, Kyustendil	Nasal swab	outbreak confirmed	Cattle	Yes	1,127
101	27 Apr 2016	Belovitsa, Hisarya, Plovdiv	Blood	outbreak confirmed	Cattle	Failed	Not sequenced
102	5 May 2016	Trilistnik, Plovdiv	Blood	outbreak confirmed	Cattle	Yes	832
115	6 May 2016	Voden, Haskovo	Blood	outbreak confirmed	Cattle	Yes	1,127 (1 N)
116	19 May 2016	Rodopi, Plovdiv	Blood	outbreak confirmed	Cattle	Yes	1,129
119	26 May 2016	Gotse Delchev area, Blagoevgrad	Blood	outbreak confirmed	Cattle	Yes	1,000

Note: Values in the final column are counts of non-gap nucleotide characters in the archived final aligned FASTA file for the five Bulgarian sequences retained in the analysis; they describe retained alignment coverage and are not PCR product sizes. The final comparative alignment comprises 1,131 nucleotide positions. One ambiguous nucleotide (N) is present in sample 115.

Supplementary Table S2. Annotated dataset of LSDV RPO30 reference sequences used in the comparative analysis

	Accession	Strain / isolate	Host	Country	Year	Virus species	Strain status
1	MH893760.2	LSDV/Russia/Dagestan/2015	Cattle (Bos taurus)	Russia	2015	LSDV	NR
2	MN072619.1	Kenya	Cattle (Bos taurus)	Kenya	NR*	LSDV	NR
3	MN995838.1	pendik	Cattle (Bos taurus)	Turkey	NR	LSDV	NR
4	MT134042.1	LSDV/Russia/Udmurtiya/2019	Cattle (Bos taurus)	Russia (Udmurtiya)	2019	LSDV	Recombinant vaccine-like strain
5	MT643825.1	BUL2016	Cattle (Bos taurus)	Bulgaria	2016	LSDV	NR
6	ON010590.1	Neethling-RIBSP/7C**	Cattle (Bos taurus)	Kazakhstan	2019	LSDV	Vaccine strain clone (Neethling-RIBSP/7C)
7	OR134832.1	LSDV/Albania/1000/2017	Cattle (Bos taurus)	Albania	2017	LSDV	NR
8	OR134833.1	LSDV/Albania/1573/2016	Cattle (Bos taurus)	Albania	2016	LSDV	NR
9	OR134834.1	LSDV/Albania/1707/2016	Cattle (Bos taurus)	Albania	2016	LSDV	NR
10	OR134835.1	LSDV/Albania/4192/2016	Cattle (Bos taurus)	Albania	2016	LSDV	NR
11	OR134836.1	LSDV/Albania/4770/2016	Cattle (Bos taurus)	Albania	2016	LSDV	NR
12	OR134837.1	LSDV/Albania/790/2017	Cattle (Bos taurus)	Albania	2017	LSDV	NR
13	OR134839.1	LSDV/Greece/386-16/2016	Cattle (Bos taurus)	Greece	2016	LSDV	NR
14	OR134840.1	LSDV/Greece/478/2016	Cattle (Bos taurus)	Greece	2016	LSDV	NR
15	OR134841.1	LSDV/Greece/498A/2016	Cattle (Bos taurus)	Greece	2016	LSDV	NR
16	OR134842.1	LSDV/Greece/715/2015	Cattle (Bos taurus)	Greece	2015	LSDV	NR
17	OR134843.1	LSDV/North_Macedonia/5000/2016	Cattle (Bos taurus)	N. Macedonia	2016	LSDV	NR
18	OR134845.1	LSDV/Serbia/4592/2016	Cattle (Bos taurus)	Serbia	2016	LSDV	NR
19	OR134847.1	LSDV/Serbia/6040/2016	Cattle (Bos taurus)	Serbia	2016	LSDV	NR
20	OR134849.1	LSDV/Serbia/7695/2016	Cattle (Bos taurus)	Serbia	2016	LSDV	NR
21	OR797612.1	LSDV/CHINA/Tibet/2023	Bovine	China (Tibet)	2023	LSDV	NR
22	PQ878211.1	LSDV_Bhutan_03	Cattle (Bos taurus)	Bhutan	2023	LSDV	NR
23	PX492334.1	155920/2012	Cattle (Bos taurus)	Belgium	NR	LSDV	NR
24	PX870246.1	LSDV_Madaripur-02-MGH-BD	Bovine	Bangladesh	2025	LSDV	NR
25	PX870247.1	LSDV_Rangpur-05-MGH-BD	Bovine	Bangladesh	2025	LSDV	NR

*NR - Not reported or not source-supported in the available GenBank record/associated publication used for annotation.

**Neethling-RIBSP/7C is a clone of the Neethling-RIBSP vaccine strain (INSDC MT130502). Strain status was assigned only when explicitly supported by the corresponding GenBank record or associated publication.

Table S3. Haplotype and sequence-based cluster assignment of RPO30 sequences included in this study.

Haplotype	N	Cluster	Samples
H1	1	C1	102
H2	1	C2	115
H3	1	C2	116
H4	1	C3	119
H5	1	C2	74
H6	1	C2	MH893760.2
H7	2	C2	MN072619.1; MT134042.1
H8	18	C2	MN995838.1; MT643825.1; ON010590.1; OR134832.1; OR134833.1; OR134834.1; OR134835.1; OR134836.1; OR134837.1; OR134839.1; OR134840.1; OR134841.1; OR134842.1; OR134843.1; OR134845.1; OR134847.1; OR134849.1; PX492334.1
H9	2	C2	OR797612.1; PQ878211.1
H10	2	C2	PX870246.1; PX870247.1

Haplotype numbering reflects the final 30-sequence dataset used in the revised analysis; the two successfully sequenced samples excluded from the final phylogenetic dataset are not included in this table.
N denotes the number of sequences assigned to each haplotype. Haplotype identifiers H1–H10 correspond to unique SNP profiles in the final 30-sequence alignment, and C1–C3

denote descriptive sequence-based clusters derived from hierarchical clustering. No separate haplotype network was constructed.

Supplementary Table S4. Polymorphic nucleotide positions within the shared aligned RPO30 region of the five Bulgarian LSDV sequences.

Alignment positions are numbered according to the multiple-sequence alignment used for the comparative RPO30 analysis. Only polymorphic positions within the region shared by all five Bulgarian sequences are shown. Sequences 115 and 116 have identical nucleotide states at all listed internal polymorphic positions. Because these are alignment-relative positions and an unambiguous codon/reference-coordinate mapping was not available for every retained sequence, synonymous/nonsynonymous effects and overlap with primer-binding sites were not assigned.

Alignment position	74	102	115	116	119
77	A	C	T	T	T
81	T	A	T	T	T
82	A	T	A	A	A
84	C	T	T	T	T
85	T	C	C	C	C
86	C	C	T	T	T
105	T	G	T	T	T
160	G	C	G	G	G
273	T	C	T	T	T
372	T	C	T	T	T
452	T	T	T	T	G
561	T	T	T	T	G
667	T	T	T	T	A
798	T	T	T	T	G
801	T	T	T	T	C
803	T	T	T	T	A
